# Polyploid Giant Cancer Cells Generated from Human Cytomegalovirus-Infected Prostate Epithelial Cells

**DOI:** 10.3390/cancers15204994

**Published:** 2023-10-15

**Authors:** Fidaa Bouezzedine, Ranim El Baba, Sandy Haidar Ahmad, Georges Herbein

**Affiliations:** 1 Pathogens & Inflammation/EPILAB Laboratory, EA 4266, University of Franche-Comté, 25000 Besançon, France; fidaa.bou_ezzeddine@univ-fcomte.fr (F.B.); ranim.elbaba@univ-fcomte.fr (R.E.B.); sandy.haydar_ahmad@univ-fcomte.fr (S.H.A.); 2 Department of Virology, CHU Besançon, 25030 Besançon, France

**Keywords:** human cytomegalovirus, CIN, PGCCs, high-risk HCMV strains, prostate cancer, oncogenesis, stemness

## Abstract

**Simple Summary:**

Prostate cancer remains a leading cause of death in men worldwide. Polyploid giant cancer cells (PGCCs) and chromosomal instability have been proposed to drive the progression of cancer. Given that HCMV infection has been implicated in malignant diseases from different cancer entities, in the present study, we assessed its transformation potential in vitro and evaluated the obtained cellular and molecular phenotypes of prostate epithelial cells (PECs) using HCMV high-risk clinical strains, DB and BL, which were previously isolated in our laboratory. HCMV-induced PGCC formation, Myc and EZH2 upregulation, as well as the stemness and epithelial–mesenchymal transition features verified the transformation process of PECs. Our research work deserves to be distributed among the scientific community, as it paves the way for upcoming studies targeting the potential role of HCMV and PGCCs in prostate cancer development and treatment.

**Abstract:**

Background: Prostate cancer is the most commonly diagnosed malignancy and the sixth leading cause of cancer death in men worldwide. Chromosomal instability (CIN) and polyploid giant cancer cells (PGCCs) have been considered predominant hallmarks of cancer. Recent clinical studies have proven the association of CIN, aneuploidy, and PGCCs with poor prognosis of prostate cancer (PCa). Evidence of HCMV transforming potential might indicate that HCMV may be involved in PCa. Methods: Herein, we underline the role of the high-risk HCMV-DB and -BL clinical strains in transforming prostate epithelial cells and assess the molecular and cellular oncogenic processes associated with PCa. Results: Oncogenesis parallels a sustained growth of “*CMV-Transformed Prostate epithelial cells*” or CTP cells that highly express Myc and EZH2, forming soft agar colonies and displaying stemness as well as mesenchymal features, hence promoting EMT as well as PGCCs and a spheroid appearance. Conclusions: HCMV-induced Myc and EZH2 upregulation coupled with stemness and EMT traits in IE1-expressing CTP might highlight the potential role of HCMV in PCa development and encourage the use of anti-EZH2 and anti-HCMV in PCa treatment.

## 1. Introduction

Prostate cancer (PCa) is the most common solid tumor in men and the most prevalent tumor in the genitourinary system [1]. Based on the American Cancer Society’s statistics in 2022, PCa was considered the most frequent malignancy and the second-ranked cause of death among men in the United States [2,3]. Variable risk factors may influence the risk of developing PCa; however, little evidence exists for PCa prevention strategies except for early diagnosis, which aids in reducing PCa mortality [2,3]. In asymptomatic early stages, PCa, also called localized PCa, is not detectable. This explains the severity of PCa in terminal stages, indicating the presence of metastatic sites or a castration-resistant cancer form (CRPC), which are often difficult to treat. At that stage, the median survival is 9 to 30 months [4].

Chromosomal instability, or CIN, has long been viewed as a topic of choice in cancer research [5] and a predominant hallmark of cancer [6]. Studying CIN can be beneficial for patient management involving diagnosis, prognosis, therapy, and genetic counseling [7]. A study showed that therapy monitoring using CIN led to the reduction of genome chaos-mediated drug resistance [5]. It is worth noting that CIN and aneuploidy are not identical, as aneuploidy represents a state of imbalanced karyotype [8]. In PCa, clinical studies revealed that CIN and aneuploidy have been linked to aggressive lethal disease [8,9], where castration and chemotherapy-resistant tumors showed a high incidence of CIN [9]. Polyploid giant cancer cells, or PGCCs, are formed through endoreplication and are considered a special subpopulation of cancer cells that contribute to solid tumor heterogeneity [10,11]. Recently, urinary PGCCs were detected in prostate cancer tissues [12]. Further, these giant cells contributed to the production of mononucleated aneuploid cells via neosis and might result in clinical relapse and chemoresistance in CRPC [13].

Prostate cancer pathogenesis involves heritable and environmental factors. A possible viral etiology of prostate cancer progression has been suggested, and evidence for viral-mediated genetic changes and associated immune dysregulation has been explored for many viruses, especially those known for infection of the anogenital and urinary sites [14]. HCMV, or human herpesvirus 5 (HHV-5), is a ubiquitous DNA virus that is detected in the urine and prostate tissue of infected subjects [15,16]. It infects between 40% and 95% of the population worldwide [17]. In healthy individuals, HCMV infection is considered mild or asymptomatic; however, in immunocompromised patients, it causes life-threatening illness in addition to congenital infections [18]. HCMV of prostatic origin established a long-term persistent infection in human embryo lung cells (HEL cells) in vitro [19]. Further, HCMV-specific intracellular immunofluorescent antigens were detected in prostatic cancer cell cultures [20,21]. An HCMV isolate that transformed HEL cells in vitro was yielded from normal human prostatic tissue [21]. Upon transplantation to athymic nude mice, these cell transformants were highly oncogenic [20]. These complex findings constitute further evidence of HCMV transforming capacity and indicate that HCMV may be involved in the development of prostatic neoplasia [20,21,22].

In agreement with the research findings of Rapp and Geder around fifty years ago, beyond the oncomodulation model, the presence of HCMV nucleic acid and proteins in several tumors (for instance, glioblastoma, neuroblastoma, colon cancer, breast cancer, and ovarian cancer) promoted HCMV as an oncovirus and highlighted a strong link between persistent HCMV infection and malignancy [23]. Recently, our group confirmed a potential direct oncogenic feature of HCMV and highlighted the role of polyploid giant cancer cells (PGCCs) in the HCMV-mediated transformation process, in agreement with the giant cell cycling theory unveiled recently by Liu and colleagues [24,25]. The oncogenic processes associated with HCMV infection at the cellular and molecular levels involved the transformation of epithelial cells, sustained proliferative signaling, growth suppressor evasion and apoptosis, and generation of PGCCs, as well as invasion and metastasis [26,27,28].

In line with the oncogenic potential of HCMV in mammary epithelial cells, we investigated its link with PCa. First, by revealing a direct transformation of prostate epithelial cells infected by the high-risk HCMV clinical strains (DB and BL HCMV strains) already isolated in our laboratory. Second, by exploring the molecular and cellular oncogenic processes associated with PCa cancer—for instance, generation of PGCCs, formation of soft agar colonies, and spheroid formation, as well as the induction of stemness and EMT markers.

## 2. Materials and Methods

**Cell cultures.** Human prostate epithelial cells (PECs) (Ref: CC-2555) were purchased from Lonza (Basel, Switzerland). The cells were maintained in prostate epithelial cell growth medium BulletKit (CC-3166) and were cultured under standard conditions (37 °C, 5% CO_2_, 95% humidity). Cultures were free of mycoplasma as determined by monthly screening (VenorGem classic mycoplasma detection, Minerva biolabs).

**Viral growth and PEC infection.** Clinical HCMV strains, namely HCMV-DB (GenBank KT959235), -BL (GenBank MW980585), -KM, and -FS, were isolated from patients who were hospitalized at Besançon University Hospital (Besançon, France) as previously described [26,29]. The human prostate epithelial cells were infected overnight with a multiplicity of infection (MOI) of 1. Screening of our viral stocks was conducted using real-time quantitative PCR to rule out the presence of other oncoviruses, namely EBV (sense, 5′-GATTTGGACCCGAAATCTGAT-3′; anti-sense, 5′-TCTGGGGGCTTATTCCTCTT-3′) and HPV (HPV16(E6) sense, 5′-GCACCAAAAGAGAACTGCAATGTT-3′; anti-sense, 5′-AGTCATATACCTCACGTCGCAGTA-3′). PEC infections, quantification of HCMV replication, and HCMV detection were performed as previously described [26]. For HCMV quantification, supernatants from infected PECs were harvested, DNA was isolated (EZNA Blood DNA Kit, D3392-02, Omega BIO-TEK, Norcross, GA, USA) and real-time IE1 quantitative PCR (qPCR) was performed using a Stratagene Mx3005P thermocycler (Agilent Technologies, Santa Clara, CA, USA) and IE1 primers (sense, 5′-CGACGTTCCTGCAGACTATG-3′; anti-sense, 5′-TCCTCGGTCACTTGTTCAAA-3′). qPCR was carried out using KAPA SYBR FAST Master Mix (KAPA BIOSYSTEMS, Potters Bar, UK). Results were collected and analyzed using MxPro qPCR software (Version 3.2).

**Microscopy.** Infected prostate cell cultures were monitored by an Olympus optical microscope (Olympus Corporation, Tokyo, Japan) and OPTIKA microscopy digital camera (Opticam, Romano d’Ezzelino, Italy). Confocal microscopy of prostate epithelial cells was performed as described previously [26]. Briefly, cells were washed with 1× PBS, fixed and permeabilized (BD Cytofix/Cytoperm, 554722) and subsequently stained with the following antibodies: anti-IE1 (pp72), anti-Myc, anti-EZH2, anti-Nestin, anti-Nanog, anti-SOX2, anti-vimentin, and anti-E-cadherin. For visualization of the nucleus and the cytoplasm, DAPI (4′, 6′-diamidino-2-phenylindole) and phalloidin staining, respectively, were performed according to the manufacturer’s protocol. After staining, confocal imaging was performed using a 63× oil immersion objective lens with an LSM800 Carl-Zeiss confocal microscope (Germany). Images were analyzed by using ZenBlue Software (Version 3.6, Carl-Zeiss Microscopy GmbH, Oberkochen, Germany). The antibodies used are provided in Supplementary Table S1.

**Flow cytometry analysis.** 1 × 10^5^ cells were collected from uninfected and infected PECs. Cells were fixed and permeabilized using 100 μL of BD Biosciences Cytofix/Cytoperm solution (554722) for 20 min at 4 °C and washed twice with 1× BD Perm wash. Cells were then stained using the respective primary and secondary antibodies, as reported previously [26]. Cells were then subjected to cytofluorometric analysis using a BD LSRFortessa X-20 (BD Biosciences, Franklin Lakes, NJ, USA) flow cytometer. Data obtained were analyzed and processed using FACSDiva software (BD FACSDiva 8.0.1, BD Biosciences). The antibodies used are provided in Supplementary Table S1.

**Soft agar colony formation assay.** Colony formation in soft agar seeded with uninfected PECs and DB- and BL-infected PECs was assayed using Cell Biolabs Cytosolic Cell Transformation Assay Kit (Colorimetric assay, CB 135; Cell Biolabs Inc., San Diego, CA, USA) as per the manufacturer’s protocol. Colonies were observed under an Olympus microscope (Olympus Corporation, Tokyo, Japan).

**Spheroid formation assay.** Spheroids of PECs were prepared as described previously [30]. Single cells (2 × 10^3^) isolated using accutase were seeded in a serum-free PEC medium containing methylcellulose.

**Statistics.** Quantitative results are reported as mean ± SD of independent experiments. Statistical analyses were done using the Mann–Whitney test; a *p*-value ≤ 0.05 was considered to be statistically significant [*: ≤0.05; **: ≤0.01; ***: ≤0.001]. Microsoft Excel v2309 was used to construct the plots and histogram data.

## 3. Results

### 3.1. HCMV-DB and -BL Clinical Strains Chronically Infected PECs Generating CTP Cells with PGCCs

**Figure 1:**
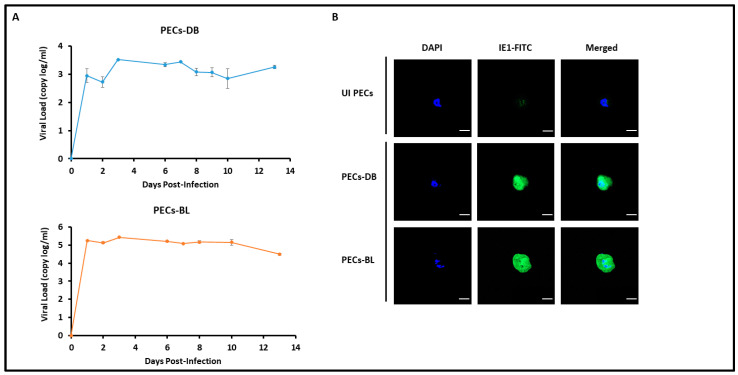
**Replication of HCMV-DB and -BL strains in PEC cultures**. (**A**) Time course of the viral titer in the supernatant of PECs infected with HCMV-DB and -BL as measured by IE1-qPCR. (**B**) Confocal microscopic images of IE1 staining in PEC-DB and -BL. UI PECs were used as controls; magnification ×63, scale bar 10 μm. Data are represented as mean ± SD of two independent experiments.

In PECs, all HCMV clinical strains replicated, showing a peak viral replication at days 3, 3, 13, and 9 post-infection with HCVM-DB, HCMV-BL, HCMV-KM, and HCMV-FS, respectively (Figure 1A and Supplementary Figure S1). The peak of HCMV load was 3 and 5 logs in PEC-DB and -BL, respectively (Figure 1A). HCMV-IE1 protein was detected in PEC-DB and -BL (Figure 1B). HCMV replication was further detected in the chronically infected PEC-DB and -BL cells (Supplementary Figure S2).

**Figure 2:**
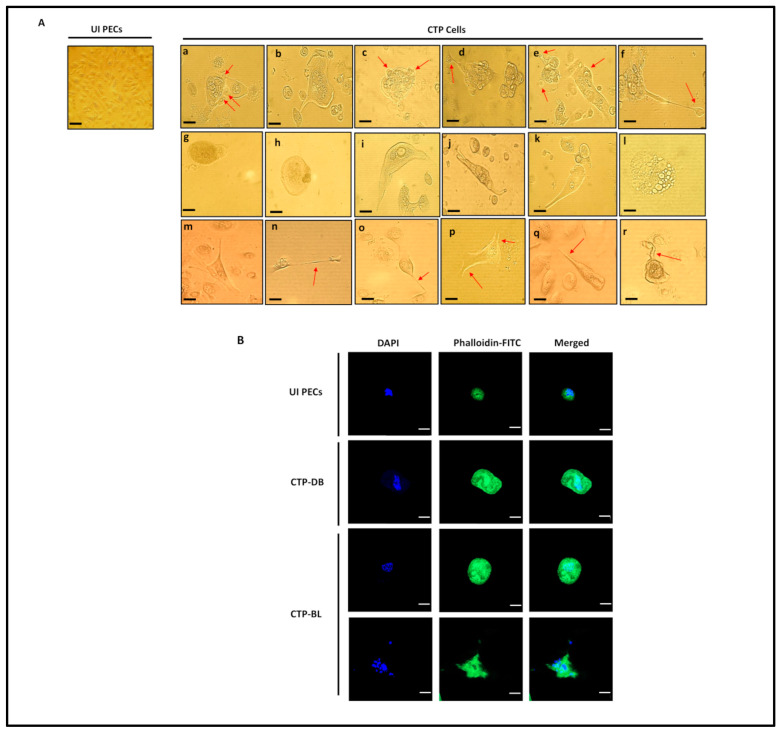
Chronic infection of PECs with the high-risk HCMV strains and detection of morphological heterogeneity in CTP cultures. (**A**) Microscopic images of distinct cellular morphologies of the giant cell cycle, including (**a**–**f**) budding, (**g**,**h**) blastomeres and blastocytes, (**i**–**k**) mesenchymal cells, (**l**) lipid droplet-filled cells, (**m**–**r**) filopodia; magnification ×100, scale bar 100 μm (for UI PECs) and ×200, scale bar 50 μm (for images **a**–**r**). Uninfected PECs were used as a control. (**B**) Confocal microscopic images of DAPI and phalloidine staining in CTP-DB and -BL cells. Uninfected PECs were used as a negative control; magnification ×63, scale bar 10 μm. Red arrows show the defined morphologies.

**Figure 3:**
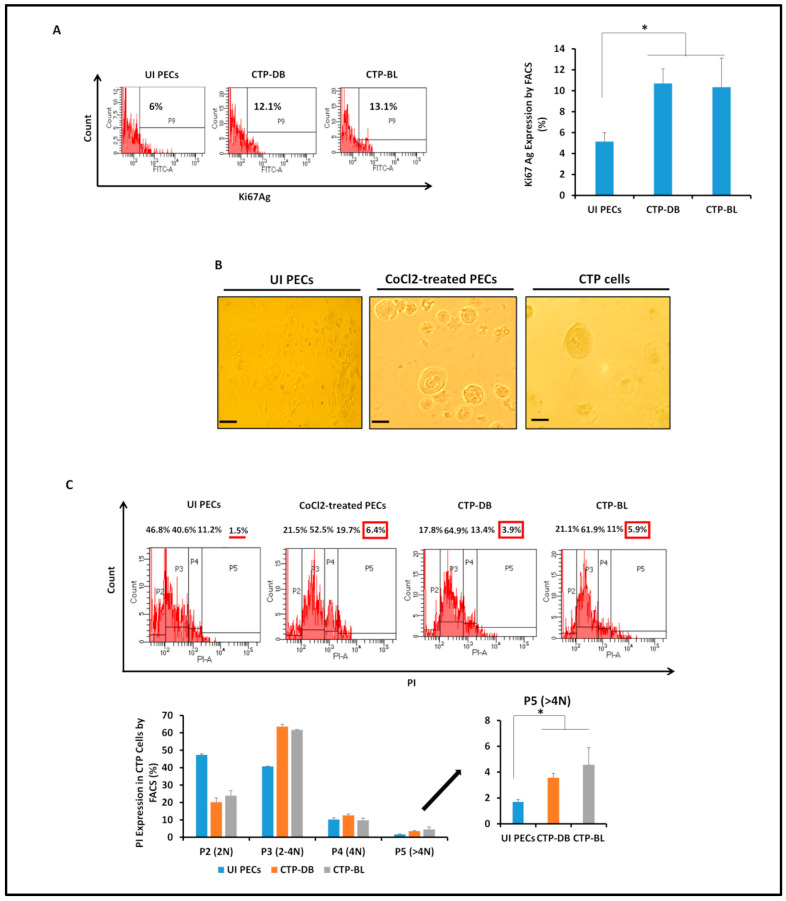
**Assessing the proliferative potential and detecting polyploidy in CTP-DB and -BL cultures**. (**A**) FACS staining of Ki67Ag in uninfected PECs as well as CTP-DB and -BL cells. (**B**) Microscopic images of polyploidy detected in CTP-DB and -BL cultures. Cobalt chloride (CoCl_2_)-treated PECs (300 μM) were used as a positive control, while uninfected PECs were used as a negative control. Magnification ×200, scale bar 50 μm. (**C**) Propidium iodide (PI) staining for polyploidy detection in CTP-DB and -BL cells by FACS. CoCl2-treated PECs were used as a positive control, and uninfected PECs were used as a negative control. Data are represented as mean ± SD of two independent experiments. * *p*-value ≤ 0.05. The red line shows the low percentage of P5 (>4 N). Red boxes emphasizes the high percentages of p5 (>4 N).

In chronically infected PEC cultures, we detected giant cells having large nuclei that were observed only in PECs chronically infected with the high-risk HCMV-DB and -BL strains, unlike uninfected PECs (Figure 2A) and PECs chronically infected with HCMV-KM and -FS strains. The emerging cells were termed “*CMV-Transformed Prostate epithelial cells*” or CTP cells, equivalent to the transformed cells that were previously reported by our group, namely, “CMV-Transformed Human mammary epithelial cells” or CTH cells, and “CMV-Elicited GlioBlastoma Cells” or CEGBCs [28,29]. Cellular heterogeneity, including giant cells, blastomeres, blastocytes, multinucleated, budding, and mesenchymal cells, as well as cells showing filopodia and lipid droplets (Figure 2A), were detected in CTP-DB and -BL cultures. The presence of PGCCs and budding cells was confirmed by confocal staining of CTP cells (Figure 2B). On evaluating the proliferation potential of CTP cells, Ki67Ag was assessed in CTP cells; Ki67Ag expression was elevated in CTP-DB and -BL cells compared to uninfected PECs (*p*-value _(UI PECs:CTP-HCMV)_ = 0.02) (Figure 3A). Further, polyploidy was detected mainly in CTP-DB and -BL cells compared to UI PECs; cobalt chloride (CoCl_2_) was used as a positive control to induce PGCC formation in PEC cultures (Figure 3B). CTP-DB and -BL cell populations were classified into PGCCs (>4 N), large cells (4 N), intermediate cells (ICs of 2–4 N), and small cells (SCs of 2 N) (Figure 3C). The percentage of PGCCs was significantly increased in CTP-DB and -BL cultures (*p*-value _(UI PECs:CTP-HCMV)_ = 0.02). Increased polyploidy observed in CTP-DB and -BL cultures is in line with an almost two-fold increase in the S-phase (2–4 N) cells compared to uninfected PECs. Further, a drastic two-fold reduction was noticed in the 2 N cells of CTP-DB and -BL cultures compared to uninfected PECs (Figure 3C). Altogether, increased DNA synthesis and polyploidy were observed in CTP-DB and -BL cells compared to uninfected PECs.

### 3.2. CTP Cells Exhibited Dedifferentiation, Stemness, and EMT Traits Parallel to the Sustained HCMV Replication

**Figure 4:**
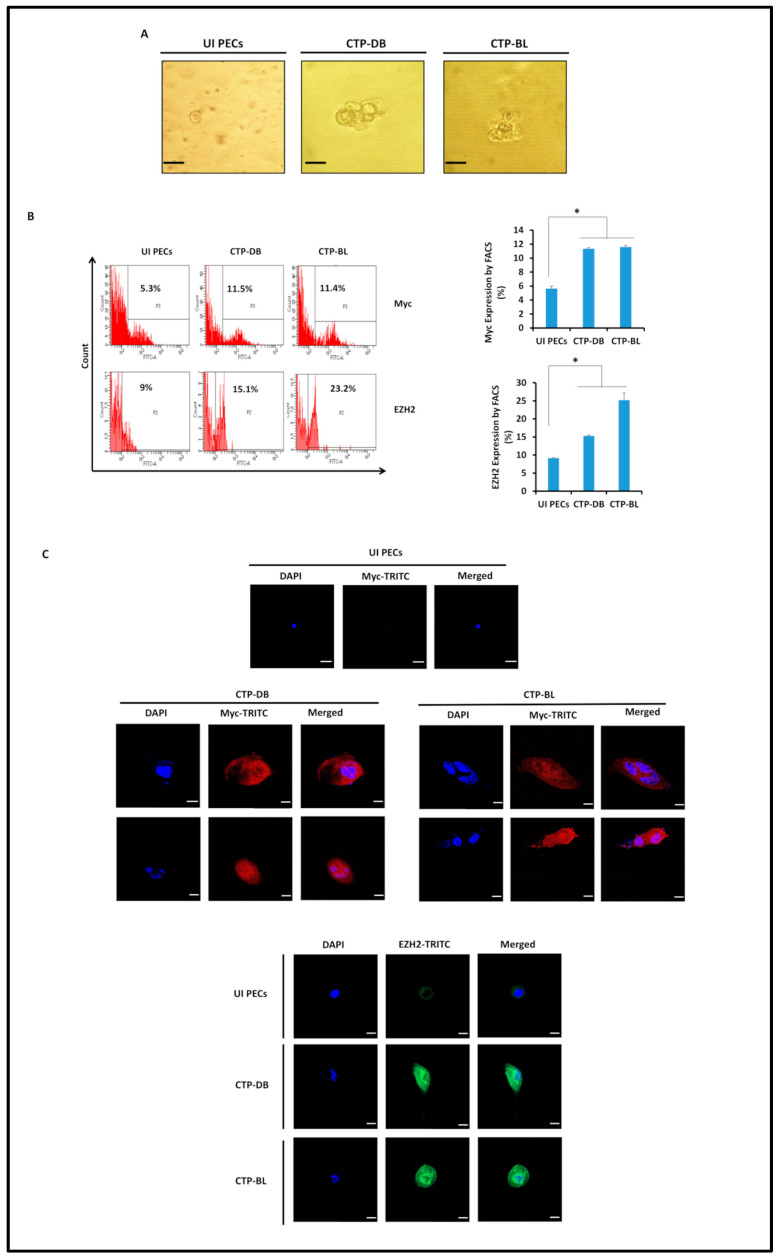
**Transformation potential and phenotypic characterization of CTP-DB and -BL cells.** (**A**) Colony formation in soft agar seeded with CTP-DB and -BL cells; uninfected PECs were used as a negative control. Formed colonies were observed under an inverted light microscope (magnification 200×, scale bar 50 µm). (**B**) FACS staining of Myc and EZH2 in uninfected PECs as well as CTP-DB and -BL cells. Data are represented as mean ± SD of two independent experiments. (**C**) Confocal microscopic images of Myc, EZH2, and DAPI staining in CTP-DB and -BL cells. Uninfected PECs were used as controls; magnification ×63, scale bar 10 μm. * *p*-value ≤ 0.05.

CTP-DB and -BL cells were seeded in soft agar to assess their oncogenic transforming potential. Colony formation was detected in the cultures seeded with CTP-DB and -BL cells compared to uninfected PECs (Figure 4A). EZH2 and Myc upregulation was detected in CTP-DB and -BL cells compared to uninfected PECs (*p*-value _(UI PECs:CTP-HCMV)_ = 0.02) (Figure 4B). In CTP cells, the elevated levels of EZH2 and Myc proteins were confirmed by confocal microscopy imaging (Figure 4C). Suz12 expression was decreased in CTP-DB and -BL cells compared to uninfected PECs (*p*-value _(UI PECs:CTP-HCMV)_ = 0.02) (Supplementary Figure S3).

**Figure 5:**
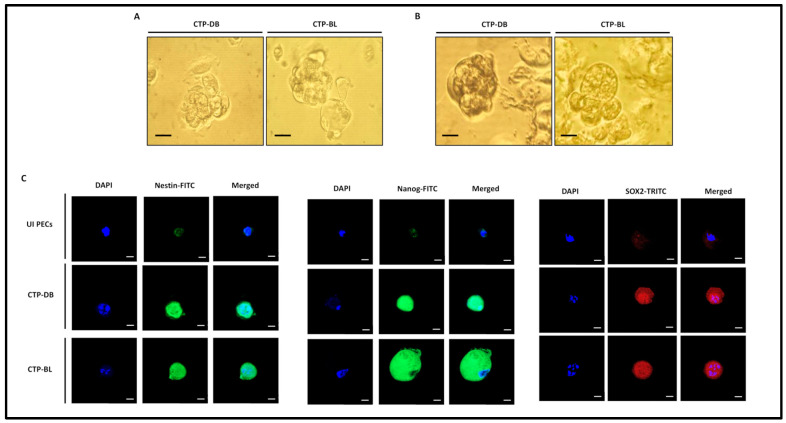
**CTP-DB and -BL cells display a stemness phenotype.** (**A**) Spontaneous spheroids were detected under an inverted light microscope in CTP-DB and -BL cultures. Magnification ×200, scale bar 50 μm. (**B**) Spheroids were observed in the chronically infected CTP-DB and -BL cell cultures in methylcellulose, scale bar 25 μm. (**C**) Confocal microscopic images of Nestin, Nanog, SOX2, and DAPI staining in CTP-DB and -BL cells. Uninfected PECs were used as controls; magnification ×63, scale bar 10 μm.

In addition to PGCC presence in CTP cultures, spontaneous spheroids, as well as methylcellulose-induced spheroids, were detected in CTP-DB and -BL cultures (Figure 5A,B). Nestin, an intermediate filament protein and marker of undifferentiated cells [31], was upregulated in CTP-DB and -BL cultures compared to uninfected PECs (Figure 5C). Nanog and SOX2 expression in CTP-DB and -BL cells revealed an embryonic stemness phenotype within the aforementioned HCMV-transformed cells (Figure 5C).

**Figure 6:**
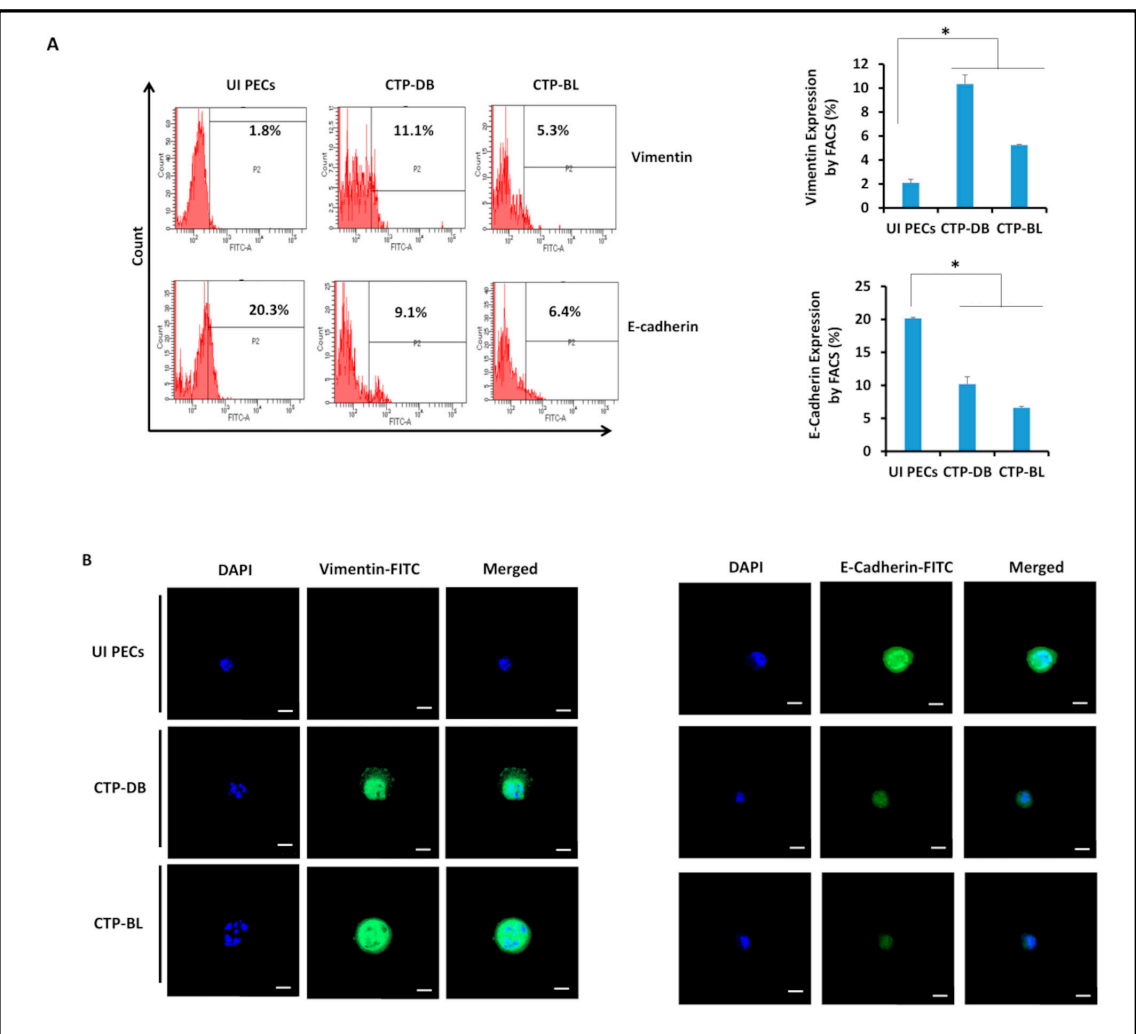
**EMT in CTP-DB and -BL cultures.** (**A**) Vimentin and E-cadherin expression by FACS in CTP-DB and -BL cells. Uninfected PECs were used as controls. Data are represented as mean ± SD of two independent experiments. (**B**) Confocal microscopic images of vimentin, E-cadherin, and DAPI staining in CTP-DB and -BL cells. Uninfected PECs were used as controls; magnification ×63, scale bar 10 μm. * *p*-value ≤ 0.05.

Through EMT, tumors can attain a mesenchymal phenotype close to cancer stem cell features that contribute to metastasis [32]. Vimentin expression was upregulated in CTP-DB and -BL cultures (*p*-value _(UI PECs:CTP-HCMV)_ = 0.02), whereas E-cadherin was downregulated compared to uninfected PECs (*p*-value _(UI PECs:CTP-HCMV)_ = 0.02) (Figure 6A,B).

**Figure 7:**
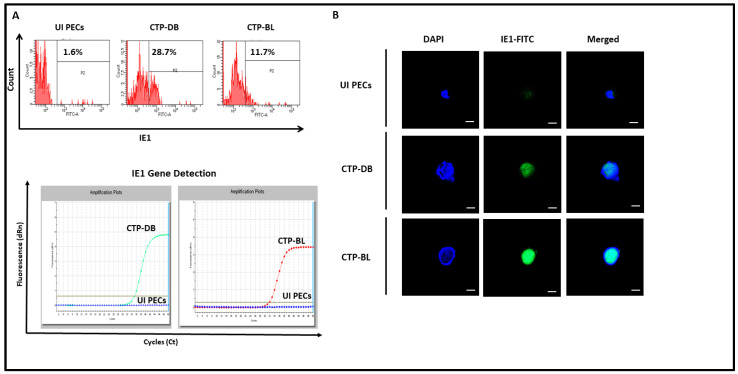
**Sustained HCMV replication in CTP-DB and -BL cultures.** (**A**) IE1 protein and gene expression in chronically infected CTP-DB and -BL cell cultures by FACS and qPCR, respectively. Uninfected PECs were used as a negative control. (**B**) Confocal microscopic images of IE1 and DAPI staining in CTP-DB and -BL cells. Uninfected PECs were used as controls; magnification ×63, scale bar 10 μm.

In PECs chronically infected with high-risk HCMV-DB and -BL, HCMV replication was sustained (Figure 7). IE1 protein and gene were remarkably detected in CTP-DB and -BL cells versus uninfected PECs (Figure 7A,B).

## 4. Discussion

**Figure 8:**
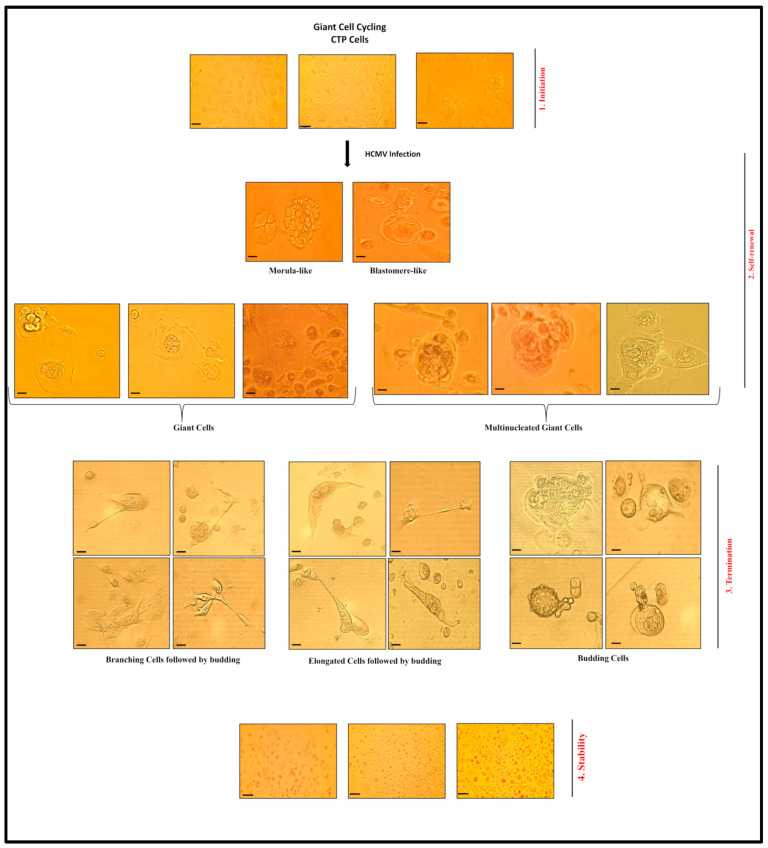
**A schematic diagram illustrating giant cell cycling in CTP-DB and -BL cultures.** Microscopic images of distinct cellular morphologies of the giant cell cycle; magnification ×200, scale bar 50 μm (initiation, self-renewal, and termination phases), and ×100, scale bar 100 μm (stability phase). Initiation, self-renewal, termination, and stability represent the four different stages of the giant cell cycle. Post-HCMV infection and via endoreplication, the 2 N PECs go into the initiation phase. Subsequently, polyploid cells (>4 N) and tetraploid cells (4 N) are produced in the self-renewal/dedifferentiation phase. During the termination/differentiation stage, the intermediate cells (2–4 N) will be generated from multinucleated or mononucleated giant cells through budding. Intermediate PECs enter the stability stage and are afterward replaced by diploid small PECs (2 N).

Polyploidy, a feature of chromosomal instability, can be triggered by oncoviruses [33]. In our study, we evaluated the transforming capacities of the high-risk oncogenic HCMV-DB and -BL strains following PEC infection. An oncogenic environment, along with a sustained growth of CTP cells with soft agar colonies, was detected upon infecting PECs with HCMV-DB and -BL. The HCMV-transformed PECs or CTP cells dedifferentiated, displayed stemness traits, gained mesenchymal features promoting EMT, and yielded PGCCs (Figure 8 and Supplementary Figure S4), and they also possessed spheroid formation potential. HCMV detection, PGCC appearance, and Myc and EZH2 upregulation verified the transformation process in PECs.

CIN/aneuploidy is associated with tumor progression and is considered a poor prognostic biomarker in various tumor types, including prostate cancer [8,9]. Knowing that the exerted potential oncomodulatory effects of HCMV infection are through enhanced replication stress, subverted DNA damage response, and induced genomic instability [34], it is of high importance to maintain CIN/aneuploidy under a tolerable threshold; this is to prevent tumor aggressiveness [7,9]. PGCC presence has been described in multiple tumors—for instance, breast, ovarian, colon, melanoma, lung, pancreas, urinary bladder, renal, thyroid, and prostate [24,26,35,36]. Studies with human prostate cancer cell lines showed that PGCCs are more aggressive, metastatic, and highly resistant to common chemotherapies [25,37]. They constitute stem cell-like traits and express various embryonic stem cell markers facilitating cancer cell survival, therapy resistance, and tumor relapse [36,37]. In line with the previous studies, CTP cells showed various cellular morphologies, including multinucleated cells, cell budding, blastomeres, blastocytes, filopodia, and cells with lipid droplets. The aforementioned morphologies were previously detected in PCa [36,38,39,40]. In agreement with the blastomere-like stemness and giant cell life cycle reported previously by Niu et al. [41,42], we observed the PGCC appearance as well as cellular heterogeneity in HCMV-transformed prostate epithelial cell cultures (CTP cells). The CTP cell population was highly heterogeneous, including PGCCs, blastomere-like cells, morula-like cells, mesenchymal cells, and small cells. The described patterns could be representative of self-renewing cells undergoing diverse stages of the previously described giant cell cycle. Thus, our outcomes revealed that PGCCs harboring HCMV might promote a malignant phenotype via giant cell cycling [24,25].

In addition to PGCCs, numerous other cellular and molecular mechanisms could account for the genome chaos observed in various solid tumor types, including, among others, chromothripsis [43], drug-tolerant persister (DTP) cancer cells [44], reversible senescence [45], treacherous apoptosis [46], oncogenic p21WAF1 expression [47,48], and anastasis [49]. Although we identified here the role of HCMV in PGCC formation, we cannot exclude the role of HCMV in the appearance of other aspects of genome chaos. HCMV induces the generation of intracellular reactive oxygen intermediates (ROIs) within minutes after infection of the cell, and then it uses these ROIs to facilitate its own gene expression and replication. Conversely, antioxidants inhibit HCMV immediate early gene expression and viral replication [50]. Since there is an increasing consensus that disrupting redox homeostasis by intervening with redox signaling is theoretically a promising therapeutic strategy for targeting drug-tolerant persister cancer cells [51], it might be possible that HCMV will modulate redox signaling in infected cells and, thereby, play a role in the generation/maintenance of DTP cancer cells. Programmed cell death or apoptosis has generally been viewed as a protective mechanism that suppresses tumor growth and prevents tumor progression. However, recent studies have shown that apoptosis can also have a paradoxical pro-tumoral role by increasing genomic instability, creating an immunosuppressive microenvironment, or driving therapy resistance. Thus, recently, it has been shown that islands of apoptotic cell death are strongly associated with tumor heterogeneity in their special proximity in vivo and induce therapy resistance in vitro. Since apoptosis disorder is a key pathogenesis mechanism of HCMV-related diseases [52], we cannot exclude that HCMV infection could be involved in the foci of apoptotic cells, thereby leading to tumor heterogenicity/therapy resistance.

Recent advances in genomics and proteomics highlighted the essential role of certain biomarkers in several tumors, mainly PCa [53,54]. Myc overexpression contributed to prostate tumor initiation and progression by disrupting transcriptional pause release at androgen receptor-regulated genes [55]. Other studies highlighted the oncogenic function of the methyltransferase EZH2 in castration-resistant prostate cancer cells, where EZH2 acts as a coactivator for critical transcription factors, including the androgen receptor [56,57]. In line with the previously mentioned findings, Myc and EZH2 overexpression was detected in CTP-DB and -BL cells compared to uninfected PECs. The CTP stemness phenotype was confirmed by the spheroids detected in CTP-DB and -BL cultures in addition to the high expression of nestin, Nanog, and SOX2, which are shown to be linked to prostate cancer development, progression, invasion, and metastasis [31,58,59,60]. Prostate cancer cells acquire invasive and metastatic characteristics via EMT [61,62]. Increasing evidence indicates that EMT promotes prostate cancer metastatic progression, and it is mainly associated with increased stemness and drug resistance [63]. In our study, vimentin was highly expressed in CTP cells, unlike E-cadherin, which was downregulated in CTP-DB and -BL cells, in line with the EMT phenotype.

Several studies have revealed the presence of infectious agents in the prostate, including oncogenic viruses, such as HPV, EBV, and BK polyomavirus [64,65]. The strength of the viral etiology and the molecular events involved in the development of PCa are still unclear. Recent evidence concluded that the HPV high-risk types have a causal role in prostate cancer [66]. HPV oncogenic proteins (E6 and E7) exert their carcinogenic potential through interacting with and degrading tumor suppressors (p53 and Rb) [67]. The association between EBV and prostate cancer is still under debate [68]. Although herpes simplex virus (HSV) is not recognized as an oncovirus, a significant association between prostate cancer and serologic evidence of HSV-2 infection is probably due to a long latency period for prostate cancer progression after HSV-2 infection [69].

Infecting PECs with the high-risk HCMV strains, namely DB and BL, revealed a sustained viral replication through the detection of HCMV-IE1 in chronically infected cultures, suggesting that HCMV infection could potentially promote cancer progression. We believe that there is an alternation of productive and latent phases in the HCMV-transformed cells, as already reported for other herpes oncoviruses, namely KSHV and EBV [70,71].

In agreement with our results, several studies showed that HCMV may be directly involved in the development of prostatic intraepithelial neoplasia [16,22]. Nevertheless, the association between HCMV and PCa has been explored, in which two out of ten case-control studies revealed no significant association between HCMV and increased PCa risk [66,72,73,74,75,76,77,78]. In fact, the observed discrepancies between these studies at the molecular and serological level may depend on the differences in techniques’ sensitivity for detecting HCMV in prostate tissues. In addition to its direct oncogenic potential, an oncomodulatory effect of HCMV linked to the ability of HCMV to interfere with the transforming cell properties has been reported [79,80]. A study done by Blaheta et al. on the prostate cancer cell model (PC3) demonstrated that HCMV acts on the invasive properties of the prostatic cancerous cell line PC3, and this carcinogenic process is promoted by elevated c-Myc levels [81]. Also, androgen in the prostate could activate the HCMV major immediate early promoter (MIEP), leading to viral replication, which might contribute to oncomodulation in prostate cancers [82].

## 5. Conclusions

In conclusion, HCMV-induced Myc and EZH2 overexpression, along with the stemness and EMT cellular phenotypes in IE1-expressing PECs, led to the appearance of transformed CTP cells and might determine a significant model in the context of PCa. Furthermore, the use of anti-EZH2 and anti-HCMV therapies could open the door to new avenues that might be beneficial in the management of PCa, especially the more aggressive castration-resistant ones, such as hormone-refractory PCa with high metastatic potential.

## Data Availability

The datasets used and/or analyzed during the present study are available from the corresponding author on reasonable request.

## Supplementary Materials

The following supporting information can be downloaded at: https://www.mdpi.com/article/10.3390/cancers15204994/s1, Figure S1. Replication of low-risk HCMV strains in PECs cultures; Figure S2. Sustained HCMV replication in CTP-DB and BL cultures; Figure S3. Expression of Suz12 in CTP-DB and BL cultures; Figure S4. A schematic diagram representing the giant cell cycling in CTP-BL cell cultures; Table S1. List of antibodies.
