# Supplementary material for: Polyploid Giant Cancer Cells Generated from Human Cytomegalovirus-Infected Prostate Epithelial Cells
Source: Cancers (Basel). 2023 Oct 15;15(20):4994. doi: 10.3390/cancers15204994 (PMC10604969; doi:10.3390/cancers15204994)
Supplement: Supplementary file 1 [file cancers-15-04994-s001.zip › cancers-2609393-supplementary.pdf]

cancers

MDPI

Polyploid giant cancer cells generated from human cytomegalovirus-infected prostate epithelial cells

Supplementary Data

Supplementary Figures

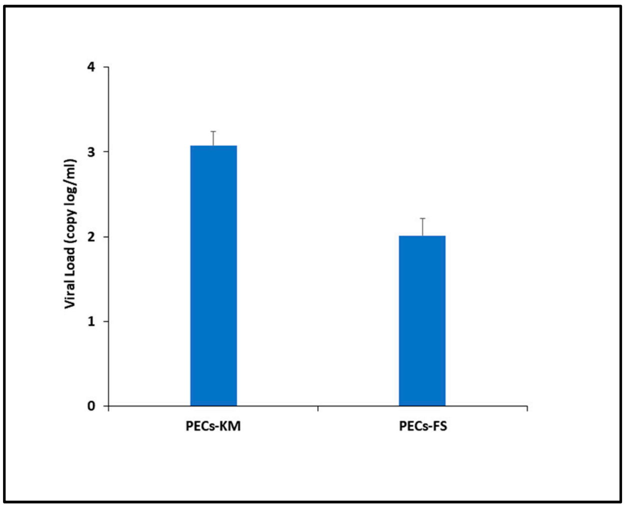

Supplementary Figure S1. Replication of low-risk HCMV strains in PECs cultures. Histogram representing the viral titer in the supernatant of PECs infected with HCMV-KM and FS as measured by IE1-qPCR.

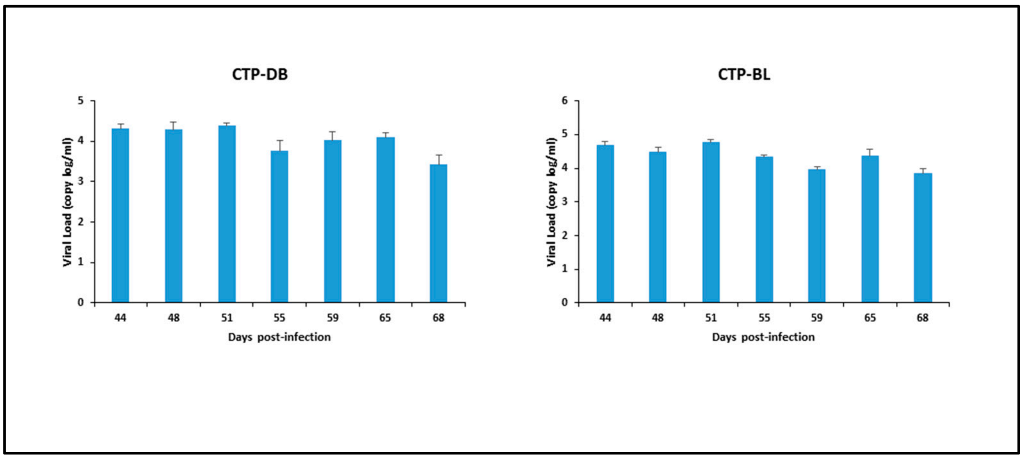

Supplementary Figure S2. Sustained HCMV replication in CTP-DB and BL cultures. Histograms representing the viral titer in the supernatant of CTP cells infected with HCMV-DB and BL as measured by IE1-qPCR. Data are represented as mean ± SD of two independent experiments.

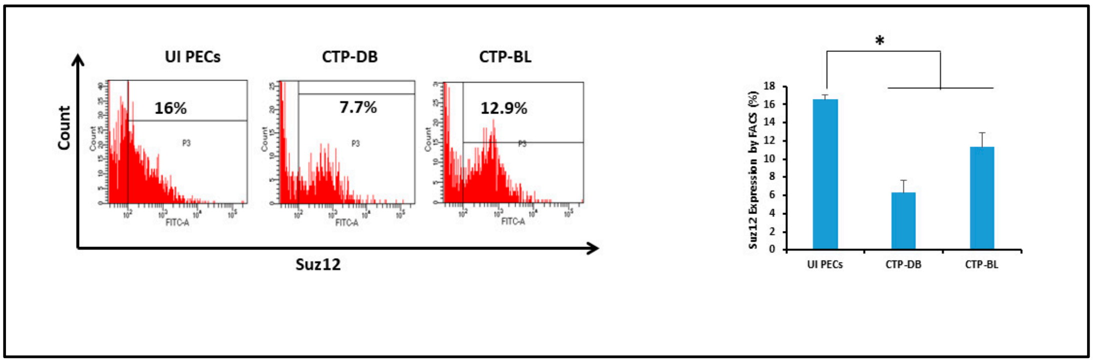

Supplementary Figure S3. Expression of Suz12 in CTP-DB and BL cultures. FACS staining of Suz12 in uninfected PECs as well as CTP-DB and BL cells. Data are represented as mean ± SD of two independent experiments. * p-value≤0.05.

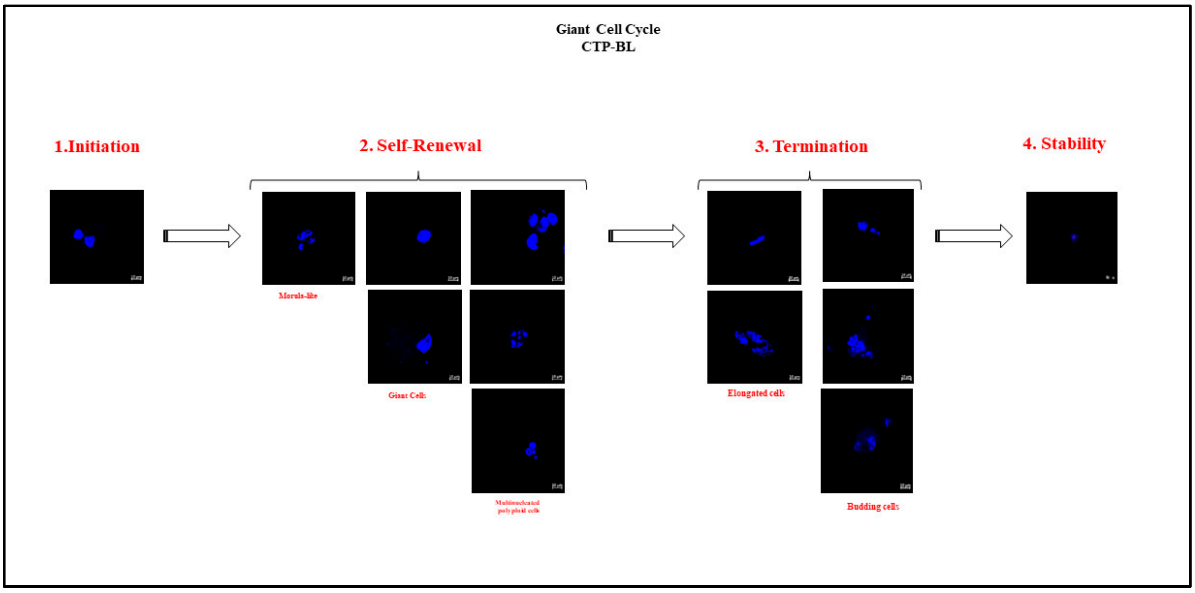

Supplementary Figure S4. A schematic diagram representing the giant cell cycling in CTP-BL cell cultures. Confocal microscopic images of DAPI staining in CTP-BL; magnification ×63, scale bar 10 μm.

Supplementary Tables

Supplementary Table S1. List of antibodies.

Antibody	Catalog Number/Source
Anti-Myc Tag	06-549-25UG/Merck KGaA, (Darmstadt, Germany)
EZH2	AB_2793397/Active Motif (Carlsbad, CA, USA)
Ki67Ag	BD-556026/BD Biosciences (Franklin Lakes, USA)
IE1	ab53495/Abcam (Cambridge, UK)
SOX2	ab97959/Abcam (Cambridge, UK)
Vimentin	SC-6260/Santa Cruz Biotechnology (CA, USA)
E-cadherin	SC-8426/Santa Cruz Biotechnology (CA, USA)
Nestin	SC-23927/Santa Cruz Biotechnology (CA, USA)
Phalloidine	ab235137/ Abcam (Cambridge, UK)
Nanog	SC-293121/Santa Cruz Biotechnology (CA, USA)
Suz12	AB_2614929/Active Motif (Carlsbad, CA, USA)
FITC-conjugated anti-mouse antibody	BD- 553399/BD Biosciences (Franklin Lakes, USA)
FITC-conjugated anti-rabbit antibody	ab6717/Abcam (Cambridge, UK)
FITC-conjugated Goat Anti-Mouse	BD-555988/BD Biosciences (Franklin Lakes, USA)
FITC-conjugated Rat Anti-Mouse	BD-553443/BD Biosciences (Franklin Lakes, USA)
Propidium Iodide	P3566/Life Technologies (Eugene, USA)
